# Dexmedetomidine attenuates acute paroxysmal sympathetic hyperactivity

**DOI:** 10.18632/oncotarget.16920

**Published:** 2017-04-07

**Authors:** Yuan Peng, Haifeng Zhu, Haodong Chen, Zijin Zhu, Huahai Zhou, Shuguang Zhang, Lili Gao, Lei Shi, Xiaoliang Li, Zhengxiang Luo

**Affiliations:** ^1^ Department of Intensive Care Unit and Neurosurgery, The First People’s Hospital of Kunshan Affiliated with Jiangsu University, Suzhou 215300, P. R. China; ^2^ Department of Neurosurgery and Medical Oncology, Jiangsu Funing People’s Hospital, Funing 224400, P. R. China; ^3^ Department of Neurosurgery, Liuhe Hospital Affiliated to Medical College of Yangzhou University, Nanjing 211500, P. R. China; ^4^ Department of Neurosurgery, Anhui Province Wangjiang Hospital, Anhui 246200, P. R. China; ^5^ Department of Neurosurgery, Sihong County People’s Hospital, Suqian 223900, P. R. China; ^6^ Department of Neurosurgery, Nanjing Brain Hospital Affiliated to Nanjing Medical University, Nanjing 210029, P. R. China

**Keywords:** dexmedetomidine, propofol, paroxysmal sympathetic hyperactivity, prognosis

## Abstract

We evaluated the curative effect of dexmedetomidine on paroxysmal sympathetic hyperactivity (PSH) in a retrospective study of 72 PSH patients after neurosurgery. Our results showed that dexmedetomidine was superior to propofol for treatment of PSH with respect to: average time needed to reduce paroxysmal hypertension (PH) to <140/90 mmHg (29.03±8.86 vs. 42.0±14.77 min), average remission time of PH (3.97±1.73 vs. 5.65±1.51 min), PH remission rate (61.22±10.8% vs. 41.52±14.15%), PH duration (9.31±2.66 vs. 13.05±4.19 days), average time for body temperature to return to normal (10.62±4.14 vs. 15.31±4.58 days), average time for heartrate to return to normal (11.34±3.90 vs. 15.72±4.10 days), and average time of respiratory rate below 25 breaths per minute (BPM) (7.00±1.74 vs. 15.32±5.87 days). Multiple logistic regression analyses showed that dexmedetomidine did not protect against the recurrence of PSH. Age and Glasgow Coma Score were the main factors predicting PSH recurrence. There was no difference in Glasgow Outcome Score (GOS) between the two groups during the 6 months of postoperative follow-up (*p*>0.05). These data suggest dexmedetomidine effectively controls an acute attack of PSH, but it does not improve the long-term prognosis of patients compared with propofol.

## INTRODUCTION

Paroxysmal sympathetic hyperactivity (PSH), also known as paroxysmal autonomic instability with dystonia (PAID), refers to the paroxysmal automatic (sympathetic nerve and parasympathetic nerve) dysfunction after spontaneous intracerebral hemorrhage and cerebral trauma [1]. PSH was first reported by Panfield in 1929; PSH patients were characterized by sudden elevation of blood pressure, increased heart rate (more than 100 beats/min), sweating, decreased or rapid (30 times/min) respiration rate, mydriasis or myosis, hiccup, and lacrimation, which is regarded as a kind of diencephalon autonomic epilepsy, opposed to ordinary epilepsy, which is caused by autonomic hyperactivity of sympathetic and parasympathetic nerves [2]. Baguley et al. [3] used the term “dysautonomia” in 1999 to systematically describe the clinical features, natural history, and clinical treatment of the disease, and the term gradually came into wide use. In 2004, Blackman et al. [1] argued that the term PAID could reflect the main features of the above syndrome, so the term was recommended. Until 2007, Rabinstein thought that the term PAID eliminated some patients with autonomic symptoms and only mild or no dystonia, so the term paroxysmal sympathetic hyperactivity (PSH) was recommended [4].

The clinical manifestations of PSH include paroxysmal autonomic and motor symptoms [5, 6]. Autonomic symptoms can be divided into sympathetic or parasympathetic hyperactivity. Sympathetic hyperactivity is characterized by hyperactivity, agitation, sweating, fever, increased heart rate and respiratory rate, hypertension, and mydriasis. Parasympathetic hyperactivity, on the other hand, is characterized by decreased heart rate and respiratory rate, decreased blood pressure, tidal breathing, lowered body temperature, and miosis; other symptoms include hiccups, tearing, and yawning. PSH can be expressed as simple sympathetic hyperactivity or combined parasympathetic and sympathetic hyperactivity. Motor symptoms include dystonia, decerebrate rigidity or decorticate posture, muscle spasm, myoclonus, and increased muscle tension. However, the main symptom of clinical cases is sympathetic hyperactivity with the manifestations of temperature change in the two directions, mydriasis, weakened senses, erected hair, tachycardia and shortness of breath. The tachycardia, fever, sweating, shortness of breath, and hypertension exist widely, but dystonia occurs in fewer than half of patients. Both autonomic symptoms and motor symptoms are paroxysmal. Any injury or other stimulus, such as the excessive sound, pain stimulation, phlegm, turning over, position change, or limb activity can induce PSH.

There is no uniform standard for PSH diagnosis. Fearnsider first proposed the diagnostic criteria for PSH in 1993 [7], after which there were eight sets of diagnostic criteria. Almost all diagnostic criteria have reached consensus on the core clinical symptoms of PSH, including the heart rate, blood pressure, respiratory rate, body temperature, sweating, and limb activity. However, there are no specific symptoms of PSH, so most scholars tend to use the exclusive method in the diagnosis. The standard proposed by Rabinstein is that patients should meet 4 out of the 6 following criteria: 1) heart rate, 2) respiratory rate, 3) body temperature, 4) high blood pressure, 5) sweating, 6) limb posture and dystonia, and body temperature was >38.3°C for 2 consecutive days. The criteria of muscle hyperactivity are the rigid posture or severe dystonia, and other causes of the above manifestations were eliminated [4]. We previously recommended the 8 criteria of Blackman: 1) severe brain injury, and cognitive function score (Rancho Los Amigos Level) <IV; 2) temperature >38.5°C; 3) pulse =130 beats/min; 4) respiratory rate >20 times/min; 5) systolic blood pressure >140mmHg; 6) excessive sweating; 7) dystonia; 8) onset times =at least 1 time/d for 3d; and the autonomic dysfunction caused by other causes was excluded [2]. However, we also tend to eliminate the autonomic dysfunction caused by other reasons without fully meeting the eight criteria of Blackman.

Due to the lack of standard and specific diagnostic criteria, the timely and targeted treatment is often lacked. At present, the treatment of PSH is mainly confined to the control of clinical symptoms [8]. Drugs used in the treatment of PSH consist of β-blockers, morphine, GABA agonists, etc [6, 9], but the effects of these drugs are often unsatisfactory, and their effect on the symptoms of paroxysmal hypermyotonia is inferior to that of propofol. Dexmedetomidine is a kind of new α2 adrenergic receptor agonist, and the patient can be awakened easily after drug administration when needed with little impact on hemodynamics and breathing, and better analgesic effect than propofol, which is the ideal sedation and analgesia drug for the severe patients and major operations [10]. Considering the advantages of dexmedetomidine, here we put forward and test the hypothesis that dexmedetomidine is better than propofol in attenuating an attack of PSH.

## RESULTS

### PSH attack aggravated the patient’s conditions

A total of 95 patients were enrolled; 32 patients in the dexmedetomidine group and 63 in the propofol group. After exclusion, there were 32 patients in the dexmedetomidine group and 40 in the propofol group, so as to ensure that there were no differences in the Glasgow Coma Scale (GCS), age and gender between the two groups before PSH attack. The severity of patients was assessed according to GCS (Table 1). The demographic and clinical characteristics of the patients are shown in Table 2. As shown in Table 2, no statistical differences of monitoring indexes, including Age, Men, and Weight, Head injury/Cerebral Hemorrhage, Hypertension history, Temperature (°C), Heart rate, Respiration rate, Blood pressure (Systolic pressure), Diabetes, Arrhythmia, or GCS were found between the dexmedetomidine group and the propofol group before PSH attack. We further analyzed the changes in monitoring parameters before and after PSH attack; GCS of these patients was basically Grade 4, and only a small number of patients were Grade 3, and we observed that there were significant changes in consciousness of patients before and after PSH attack, so we used GCS to analyze the changes in state of consciousness of patients before and after attack. As shown in Table 3, the mean temperature (°C), heart rate, respiration rate and blood pressure (systolic pressure) in PSH were significantly higher than those before PSH attack (*p*<0.05). The highest body temperature during attack was recorded as the body temperature. Moreover, GCS was decreased significantly after the attack, and it did not return to the level before attack until after the next day (specific data unavailable), indicating that PSH attack can decrease consciousness in PSH patients, PSH attack aggravates the patient’s condition, and suggests a poor prognosis for patients.

**Table 1:** Grading criteria for patients enrolled in the study

Patient’s grade	Glasgow Coma Scale (GCS)
Grade 0	Glasgow coma scale score of 15
Grade 1	Glasgow coma scale score of 15
Grade 2	Glasgow coma scale score of 13-14
Grade 3	Glasgow coma scale score of 9-12
Grade 4	Glasgow coma scale score of 3-8

**Table 2:** The demographic and clinical characteristics of the patients between 2 groups before PSH attack

Characteristics	Dexmedetomidine group	Propofol group	P-value
Age(years)	50.69±10.22	53.13±10.86	0.620
Men/women	23/9	27/13	0.689
Weight (kg)	68.95±11.09	67.97±13.41	0.741
Head injury/hypertensive intracereral hemorrhage	21/11	31/9	0.264
Hypertension history	21/11	29/11	0.529
Temperature (°C)	36.41±1.81	36.73±0.40	0.269
Heart rate(beats/min)	75.75±11.57	77.30±8.49	0.514
Respiration rate	22.25±2.97	21.07±2.51	0.073
Blood pressure(Systolic pressure)(mmHg)	126.97±6.80	122.72±11.21	0.052
Diabetes	6/26	3/37	0.173
MGS-GCS			
Grade 0	0	0	
Grade 1	0	0	
Grade 2	0	0	
Grade 3	2	2	
Grade 4	30	38	1.000

**Table 3:** The demographic and clinical characteristics of the patients before and after PSH attack

Characteristics	Dexmedetomidine group (before PSH)	Dexmedetomidine group (PSH attack)	P-value	Propofol group (before PSH)	Propofol group (PSH attack)	P-value
Temperature (°C)	36.41±1.81	38.60±0.54	<0.01	36.73±0.40	38.64±0.57	<0.01
Heart rate (beats/min)	75.75±11.57	129.44±8.10	<0.01	77.30±8.49	123.70±17.69	<0.01
Respiration rate (breaths/min)	22.25±2.97	39.62±4.92	<0.01	21.07±2.51	38.4±3.86	<0.01
Blood pressure (Systolic pressure) (mmHg)	126.97±6.80	177.44±15.63	<0.01	122.72±11.21	170.92±10.11	<0.01
GCS	6.87±1.13	4.28±0.96	<0.01	7.17±0.68	4.30±0.76	<0.01

### Dexmedetomidine effectively controls the symptoms and shortens the course of a PSH attack

Table 4 shows the symptoms and improvement of course of PSH patients after applying dexmedetomidine and propofol, including the remission time of paroxysmal hypertension, remission time of paroxysmal dystonia, remission rate of paroxysmal hypermyotonia, duration of paroxysmal hypermyotonia, average time for body temperature to return to normal, average time for heart rate to return to normal, and average time for respiratory rate to return below 25 breaths/min. After a PSH attack, blood pressure often increases rapidly, but conventional treatment, including oral administration of calcium ion antagonist, ACEI/ARB, β-receptor blocker, and even the intravenous injection of labetalol or urapidil, cannot be used to relieve it. We found that after dexmedetomidine intervention, blood pressure could be effectively maintained below 140/90mmHg within 29.03 ± 8.86 min without the need for other intravenous antihypertensive drugs; 42.0 ± 14.77 min were needed for propofol intervention along with other intravenous antihypertensive drugs to maintain the blood pressure within 140/90mmHg. Paroxysmal hypermyotonia is very common in PSH patients, and sometimes causes similar symptoms to epilepsy, but diazepam injection or midazolam has poor or no effects. As shown in Table 4, dexmedetomidine could control the symptoms of paroxysmal hypertonia in PSH patients; the average remission time of hypermyotonia using the load-dose dexmedetomidine was about 3.97 ± 1.73min, and then it was maintained using the maintenance dose, while that using propofol or combined with midazolam was about 5.65 ± 1.51min. Moreover, the remission rate of paroxysmal hypermyotonia was (61.22 ± 10.82) % within 24 hours after the administration of dexmedetomidine, while that after the administration of propofol was only (41.52 ± 14.1) %. It cost (9.31 ± 2.66) days in dexmedetomidine group to realize the complete remission, namely no recurrence, of paroxysmal hypermyotonia, and (13.05 ± 4.19) days in propofol group, respectively. The average time of decline in temperature to the normal level after drug intervention of the former was (10.62±4.14) days, while that of the latter was (15.31±4.58) days; the average time of decline in heart rate to the normal level after drug intervention of the former was (11.34±3.90) days, while that of the latter was (15.72±4.10) days; the average time of recovering respiratory rate below 25times/min after drug intervention of the former was (7.00±1.74) days, while that of the latter was (15.32±5.87) days.

**Table 4:** The demographic and clinical characteristics of the patients between 2 groups after PSH attack

Characteristics	Dexmedetomidine	Propofol	P-value
Remission time of paroxysmal hypertension (min)	29.03±8.86	42.0±14.77	<0.01
Remission time of paroxysmal hypermyotonia (min)	3.97±1.73	5.65±1.51	<0.01
Remission rate of paroxysmal hypermyotonia (%)	61.22±10.82	41.52±14.15	<0.01
Duration days of paroxysmal hypermyotonia (d)	9.31±2.66	13.05±4.19	<0.01
Average time of decline in temperature to the normal level	10.62±4.14	15.32±4.52	<0.01
Average time of decline in heart rate to the normal level	11.34±3.90	15.72±4.10	<0.01
Average time of respiratory rate returning below 25 breaths/min	7.00±1.74	15.32±5.87	<0.01

### Age and GCS score were the main predictors of PSH recurrence

Through retrospective data analysis, we found that PSH symptoms still occur in some patients after the active treatment period. We performed multivariate logistic regression analysis to further analyze the related factors of PSH recurrence. As shown in Table 5, it was found that the patient’s age and GCS score were significantly correlated with the PSH recurrence (*p*<0.05). The older the age and the lower the GCS score were, the more easily PSH will reoccur. However, it was not correlated with gender, body weight, cerebral trauma or cerebral hemorrhage, whether there was intraventricular hemorrhage, whether there was a history of hypertension or diabetes, and the treatment with dexmedetomidine. Dexmedetomidine did not protect against the recurrence of PSH. Age and GCS scores were the main predictors of PSH recurrence (Age, odds ratio=1.070, 95% confidence interval= 1.006-1.138, P=0.031; GCS Scores, odds ratio=0.416, 95% confidence interval= 0.195-0.886, P=0.023).

**Table 5:** Logistic regression analysis of factors related to recurrence

Factors	OR (95% CI)	P-value
Age	1.070 (1.006-1.138)	0.031
Sex	0.712 (0.195-2.600)	0.607
Weight	1.000 (0.976-1.024)	0.990
Head injury/hypertensive intracereral hemorrhage	1.617 (0.389-6.722)	0.508
Combined thalamic injury	2.235 (0.623-8.017)	0.217
Combined ventricular hemorrhage	1.458 (0.303-7.016)	0.638
Hypertension history	0.710 (0.193-2.613)	0.607
Diabetes history	0.895 (0.118-6.776)	0.915
GCS Scores	0.416 (0.195-0.886)	0.023
Dexmedetomidine administration	0.878 (0.248-3.107)	0.840

### Dexmedetomidine did not improve the long-term prognosis of PSH patients

During the 6-month follow-up, we further evaluated whether there were differences in effects of dexmedetomidine and propofol on the long-term prognosis of PSH patients using GOS. We found that dexmedetomidine did not improve the long-term prognosis of PSH patients, although it could better control the symptoms of PSH than propofol. As shown in Table 6, in the dexmedetomidine group, 0 cases had 5 points, 0 cases had 4 points, 3 cases had 3 points, 18 cases had 2 points, and 11 cases had 1 point; in the prophadol group, 0 cases had 5 points, 0 cases had 4 points, 2 cases had 3 points, 23 cases had 2 points, and 15 cases had 1 point. The p values for statistical analysis are shown in Table 6 and demonstrate no significant difference between the two groups.

**Table 6:** Evaluation of long-term prognosis of patients between the two groups via GOS score

Group	Dexmedetomidine	Propofol	P-value
GOS 5	0	0	
GOS 4	0	0	
GOS 3	3	2	0.650
GOS 2	18	23	0.951
GOS 1	11	15	0.784

## DISCUSSION

PSH syndrome is a kind of complication after severe cerebral injury, but its pathological and physiological mechanism remains unclear. The first widely-held belief that the diencephalon discharge was the cause of PSH, known as diencephalic epilepsy, was refuted by EEG monitoring [11]. Subsequently, many scholars put forward the “punctuated theory”, that there were control centers in the brainstem, brain, and cortex, and that damage to these control centers causes the diencephalon, brain stem sympathetic lower center or pathway derepression or change in excitatory: inhibitory ratio model, release the sympathetic nerve in depressed state and increase the sympathetic nerve activity [12]. It is speculated that the lesions affect the ventricular paraventricular hypothalamic nucleus, midbrain periaqueductal gray, lateral parabrachial nucleus and lateral medulla [13]. The changes in heart rate variability and pain stimulation-related heart rate in PSH patients prove that the sympathetic nerve activity is increased and the activity is excessive in PSH patients, but there is still a lack of biological evidence directly related to this syndrome.

Studies have shown that catecholamine concentration in plasma is significantly increased during PSH attack in patients with traumatic brain injury, suggesting that the sympathetic nerve excitation is significant during PSH attack [14]. Therefore, the treatment principles for clinical drugs are as follows: inhibit the excitement of sympathetic state, including the incoming and outgoing of nerve signals, and terminal response organs. The drugs used mainly include β-blockers, GABA agonists, morphine, and so on. In theory, β-blocker is a kind of lipid-soluble drugs that can penetrate the blood-brain barrier, and effectively reduce the catecholamine levels in the circulatory system and metabolic rate in the resting state. Some studies have shown that β-blocker positively affects the majority of PSH-related clinical symptoms, such as hypertension, hyperhidrosis, hyperthermia, tachycardia, and dystonia [15]. In fact, the symptoms of a considerable number of PSH patients are not alleviated after using β-blocker. Blackman et al. [2] argued that the practical value of β-blocker for PSH is still uncertain, and further evaluation is needed. GABA agonists, such as baclofen, can activate GABA β receptors, reduce the synaptic reflex potential of spinal synapses or multiple synapses and the reflex potential between posterior spinal roots, resulting in skeletal muscle relaxation, which can reduce the frequency and severity of spasm attack, thus relieving the spasm-related clonus, pain and muscle rigidity, etc [16]. However, clinical trials have shown that oral administration of ITB has no effect on PSH. Benzodiazepines combined with GABA α receptors increase GABA inhibition in the central nervous system by stimulating the GABA receptors in the ascending reticular activation system. They are very effective as muscle relaxers with a strong effect on rages. Lorazepam, midazolam, and diazepam are often used to treat PSH [17], but they have poor effects on patients with severe paroxysmal dystonia, inferior to opioids, such as morphine. Morphine is a kind of μ-opioid receptor agonist, which can effectively terminate a PSH attack mainly via the inhibition of the central pathway and inhibition of sympathetic nerve, but it is strongly dose-dependent and highly addictive after long-term use [17].

In this retrospective study we found that PSH syndrome often cannot be diagnosed promptly or is often misdiagnosed. When patients suffer from paroxysmal hypermyotonia and tachypnea after operation, and man-machine counteraction (ventilator-assisted breathing), and their symptoms cannot be alleviated by intravenous diazepam, doctors often choose propofol as the preferred sedative. However, it has been found that propofol can also effectively control the symptoms of PSH. Propofol is a short-acting intravenous anesthetic of the alkyl acid family, which can be distributed rapidly in the body via intravenous injection and initiates the sleep state within 40 seconds with quick and smooth anesthesia. Propofol has a mild analgesic effect, which can reduce intracranial pressure, cerebral oxygen consumption, and cerebral blood flow. It can inhibit the respiratory system, causing temporary respiratory arrest and can also inhibit the circulatory system, lowering blood pressure. The mechanism of action of propofol is rapid anesthesia and the inhibition of respiratory and circulatory system, which can improve the paroxysmal hypermyotonia in PSH patients, control the hypertension, and relieve the shortness of breath.

Dexmedetomidine is an active dextro-isomer of medetomidine, characterized by its anti-sympathetic, sedative, and analgesic effects. Compared with medetomidine, dexmedetomidine has a stronger selectivity as a central α2-adrenergic receptor agonist, and is eight times more selective than clonidine [18]. α2A receptor subtypes play an important role in the main pharmacological and therapeutic effects of this product. α2A receptor exists in presynaptic and postsynaptic fractions, and its activity is mainly related to the inhibition of norepinephrine release and neuronal excitability. Dexmedetomidine inhibits norepinephrine release, lowers plasma catecholamines, and terminates pain signal conduction by stimulating the presynaptic membrane α2 receptor. Through activating the postsynaptic membrane receptor, dexmedetomidine inhibits the sympathetic nerve activity, leading to the decreased blood pressure and heart rate. Through its action on the α2 receptor in the spinal cord, dexmedetomidine can produce an analgesic effect and lead to the sedation and anxiety remission. It is a strong sedative and does not affect the patient’s sense of judgment, making dexmedetomidine an ideal sedative for patients with severe craniocerebral injury in perioperative period [19].

Goddeau et al. reported that dexmedetomidine might be a novel pharmacologic agent to aid in abrogating PSH in a case report [20]. In this retrospective study, we compared the treatment effects of dexmedetomidine and propofol on PSH syndrome, and we found that dexmedetomidine was superior to propofol in the control of paroxysmal hypertension and dystonia, shortening the average remission time and frequency of paroxysmal hypermyotonia, and recovering the body temperature, heart rate and respiration rate to the normal level. However, dexmedetomidine was not a protective factor for the recurrence of PSH. Age and GCS score were the main factors of PSH recurrence. The 6-month postoperative follow-up showed no difference in prognosis of patients between the dexmedetomidine group and the propofol group, suggesting that dexmedetomidine does not improve the long-term prognosis of PSH patients.

In conclusion, we believe that dexmedetomidine can effectively control the onset of PSH symptoms and shorten its course, but it does not improve the long-term prognosis of patients. In addition, we observed that age and GCS score are the main predictive factors of PSH recurrence.

## MATERIALS AND METHODS

### Clinical data

95 patients with craniocerebral injury admitted to various hospitals from 2012 to 2017 were selected, including 60 cases of severe cerebral trauma and 36 cases of cerebral hemorrhage. All patients received craniotomy, and other PSH was eliminated via postoperative diagnosis. 32 patients receiving dexmedetomidine alone and 40 patients receiving propofol alone were selected as the dexmedetomidine group and the propofol group. The dexmedetomidine group consisted of 32 PSH patients, including 21 cases of midbrain trauma and 11 cases of hypertensive intracerebral hemorrhage. The propofol group consisted of 40 PSH patients, including 31 cases of midbrain trauma and 9 cases of hypertensive intracerebral hemorrhage.

### Statement for human participants

All methods in this study were carried out in accordance with relevant guidelines and regulations. All experimental protocols were approved by the licensing committees of the First People’s Hospital of Kunshan, People’s Hospital of Funing Country, Liuhe Hospital Affiliated to Medical College of Yangzhou University, Anhui Province Wangjiang Hospital, Sihong Country People’s Hospital, Nanjing Brain Hospital. Informed consent was obtained from all subjects.

### Diagnostic criteria

1) severe cerebral injury and cognitive function score (Rancho Los Amigos Level) <IV; 2) intermittent elevation of body temperature >37.5°C; 3) pulse >100 times/min; 4) respiratory rate >30 times/min; 5) systolic blood pressure >140mmHg or diastolic blood pressure >100mmHg; 6) paroxysmal excessive sweating; 7) paroxysmal dystonia; 8) onset time of at least 1 time/d for 2d; 9) autonomic dysfunction caused by other reasons was eliminated. 7 of the above criteria should be met.

### Exclusion criteria

1) patients with primary brain stem injury or brain stem hemorrhage; 2) GCS score =3 points; 3) patients complicated with chronic liver, heart and arrhythmia, and diseases in kidney, lung, and other vital organs; 4) patients complicated with severe coagulation abnormalities, hemorrhagic shock, multiple organ failure; 5) patients with a history of cerebral hemorrhage or cerebral infarction within the past three months; 6) patients complicated with a history of unhealed malignant tumor; 7) patients complicated with a history of unhealed epilepsy.

### Treatment methods

Conventional symptomatic treatment was ineffective for the above symptoms in patients with severe cerebral trauma or cerebral hemorrhage. Dexmedetomidine or propofol was applied after other factors were eliminated and PSH was diagnosed definitively. Dexmedetomidine injection: Before the administration, drug was diluted to 4μg/mL in 0.9% sodium chloride solution; 2mL sample was added to 48mL 0.9% sodium chloride injection to form the 50mL solution; the solution was gently shaken and evenly mixed. The specific dose was according to the remission degree of symptoms. A loading dose of 1μg/kg and maintenance dose of 0.3-0.6μg/kg/h could be used if necessary. Propofol injection: The dose was adjusted according to the sedative depth, continuous intravenous infusion adopted a dose of 0.3-4.0mg/kg/h, and the rate of administration did not exceed 4.0mg/kg/h.

### Definitions

The complete remission of PSH was defined as symptoms returning to normal for more than five consecutive days. Remission time was calculated from the first day when symptoms returned to completely normal. PSH recurrence referred to the PSH symptom five days after the complete remission.

### Evaluation of efficacy

We retrospectively analyzed the PSH patients receiving the symptomatic treatment with dexmedetomidine or propofol and assessed the following related clinical indicators of improvement: 1) The average time of hypermyotonia remission was calculated within 24 hours after drug intervention as follows: (total time from increase to remission of hypermyotonia every time within 24 hours after drug intervention)/onset time. 2) The remission rate of paroxysmal hypermyotonia within 24 hours was calculated as follows: (average onset time within 24 hours after drug intervention/ average onset time within 24 hours before drug intervention) *100%. 3) Duration days of paroxysmal hypermyotonia after drug intervention referred to the time from the drug intervention to the complete remission. Complete remission was defined as no-onset for five consecutive days. 4) average time of decline in body temperature to completely normal after drug intervention; patients with significant infectious factors were excluded; 5) average time of heart rate returning to normal after drug intervention; 6) average time of respiratory rate returning below 25 breaths/min after drug intervention; 7) Glasgow Outcome Scale (GOS) scores evaluated at 6 month followup after injury:1, death; 2, persistent vegetative state; 3, severe disability; 4, moderate disability; 5, mild or no disability.

### Correlation analysis

We observed that consciousness was significantly decreased in patients with cerebral injury after PSH, so the correlation analysis was conducted to analyze the correlation between PSH attack and decline in GCS score. We further evaluated the differences in prognosis between PSH patients and others with the same disease. Multiple logistic regression analysis was used to assess the correlation between PSH attack and cerebral injury site.

### Statistical analysis

The data were analyzed using SPSS version 15.0 (SPSS Inc., Chicago, Illinois). Quantitative variables such as age and body temperature are expressed as the mean ± SD. We used the χ2 or Fisher analysis to test associations between categorical variables and the t-test for continuous variables. Multiple logistic regression analysis was adopted to analyze the correlation between variables. *p*<0.05 was statistically significant.

## Abbreviations

PSH: paroxysmal sympathetic hyperactivity
α_2_-AR: α_2_-adrenoceptor
GCS: Glasgow Coma Scale
PAID: paroxysmal autonomic instability with dystonia
